# Heterozygous mutation of cysteine_528_ in XPO1 is sufficient for resistance to selective inhibitors of nuclear export

**DOI:** 10.18632/oncotarget.11995

**Published:** 2016-09-13

**Authors:** Jasper Edgar Neggers, Els Vanstreels, Erkan Baloglu, Sharon Shacham, Yosef Landesman, Dirk Daelemans

**Affiliations:** ^1^ Katholieke Universiteit Leuven, Department of Immunology and Microbiology, Laboratory of Virology and Chemotherapy, Rega Institute for Medical Research, Leuven, Belgium; ^2^ Karyopharm Therapeutics, Newton, MA, USA

**Keywords:** XPO1, CRM1, SINE, CRISPR/Cas9, nuclear export

## Abstract

Exportin-1 (CRM1/XPO1) is a crucial nuclear export protein that transports a wide variety of proteins from the nucleus to the cytoplasm. These cargo proteins include tumor suppressors and growth-regulatory factors and as such XPO1 is considered a potential anti-cancer target. From this perspective, inhibition of the XPO1-mediated nuclear export by selective inhibitor of nuclear export (SINE) compounds has shown broad-spectrum anti-cancer activity. Furthermore, the clinical candidate SINE, selinexor, is currently in multiple phase I/II/IIb trials for treatment of cancer. Resistance against selinexor has not yet been observed in the clinic, but *in vitro* selection of resistance did not reveal any mutations in the target protein, XPO1. However, introduction of a homozygous mutation at the drug's target site, the cysteine 528 residue inside the XPO1 cargo-binding pocket, by genetic engineering, confers resistance to selinexor. Here we investigated whether this resistance to selinexor is recessive or dominant. For this purpose we have engineered multiple leukemia cell lines containing heterozygous or homozygous C528S substitutions using CRISPR/Cas9-mediated genome editing. Our findings show that heterozygous mutation confers similar resistance against selinexor as homozygous substitution, demonstrating that SINE resistance can be obtained by a single and dominant mutation of the cysteine_528_ residue in XPO1.

## INTRODUCTION

Human exportin 1 (XPO1), also known as chromosome region maintenance 1 protein (CRM1), is a ubiquitously expressed member of the importin-β family of nucleocytoplasmic transport proteins (also known as karyopherins). XPO1 mediates the export of many different cargo proteins from the nucleus to the cytoplasm, including tumor suppressive or growth regulatory related proteins such as nucleophosmin, p53, p21, pRb, BRCA1, APC and FOXO family proteins, but also viral proteins, such as HIV-1 Rev, are transported by XPO1 [1]. XPO1 has been considered a potential molecular target for therapeutic intervention for decades and in recent years, its overexpression has been associated with malignancy [2-6] and XPO1 has been considered a potential molecular target for therapeutic intervention for decades. Yet, only recently the first clinical validation of pharmacological inhibition of XPO1 came with the discovery of the selective inhibitor of nuclear export (SINE) compounds. SINE are orally bioavailable, optimized *N*-azolylacrylate derivative small-molecule inhibitors of the XPO1-mediated nuclear protein export [7-10]. They bind into the hydrophobic cargo-binding groove of XPO1 and covalently modify the cysteine_528_ residue through a Michael type addition, thereby preventing the interaction of cargo with XPO1. Currently, the leading candidate of the SINE, selinexor (KPT-330), is under investigation in multiple stage I/II/IIb clinical trials. Natural or acquired resistance to selinexor has not been observed in the clinic, but in an attempt to select for resistance against these compounds *in vitro*, Crochiere and colleagues treated HT-1080 cells with gradually increasing concentrations of the drug [11]. Resistance was reached only after an extensive period of 10 months of continuous exposure. Resistant cells displayed an altered expression pattern in a number of key signaling pathways, including upregulation of anti-apoptotic and downregulation of pro-apoptotic genes. These cells were also >100 fold less sensitive to SINEs and even though they showed nuclear accumulation of XPO1 cargo-proteins in resistant cells after treatment as compared to the parental cells, they did not incorporate any mutations in the cargo-binding pocket of XPO1. This hydrophobic cargo-binding cleft of XPO1, and especially the cysteine_528_ residue located in this cleft, is highly conserved amongst higher eukaryotes. However, recently, in an effort to validate the SINE-XPO1 drug-target interaction, we were able to successfully engineer the genome of Jurkat leukemic T-cells to substitute cysteine_528_, the anchor point of selinexor inside the cargo-binding pocket of XPO1, with a serine. These homozygous genome-edited XPO1^C528S^ Jurkat cells were viable and showed a >250 fold resistance to SINE compounds, demonstrating that the anti-cancer activity of SINE compounds effectively results from XPO1 inhibition [12]. In this current study, we investigate the effect of heterozygous substitutions of the XPO1 cysteine 528 residue on SINE activity *in cellulo*.

## RESULTS

### Generation of *XPO1^C528S^* knock-in mutants by CRISPR/Cas9 genome editing

**Figure 1:**
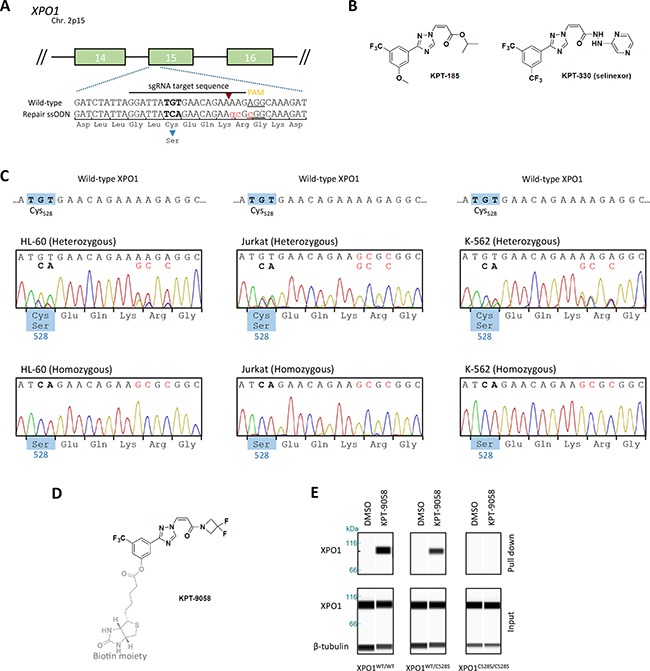
Generation of heterozygous and homozygous XPO1^C528S^ cell lines **A.** Schematic representation of the setup for CRISPR/Cas9 genome editing of *XPO1*. The numbered green boxes represent exons of *XPO1*. The wild-type sequence around cysteine_528_ located in exon 15 is enlarged. The sgRNA sequence is highlighted, its corresponding Cas9 cutting site is denoted by a red arrowhead and the PAM site is underlined. The donor oligo sequence used for homology directed repair (HDR) is shown below. The Cys528Ser missense mutation is highlighted in bold and the 3 additional silent mutations are highlighted in small red letters. **B.** Chemical structure of the selective inhibitors of nuclear export KPT-185 and KPT-330 (Selinexor). **C.** Sanger sequencing chromatograms of the generated heterozygous and homozygous XPO1^C528S^ cell lines. The desired nucleotide mutations are highlighted in bold, while silent mutations are shown in red. The amino acid residue located at position 528 is shown in the blue boxes. **D.** The chemical structure of the biotinylated selective inhibitor of nuclear export KPT-9058. The biotin moiety is highlighted in light grey and the active warhead, which binds to XPO1 is shown in black **E.** Pull down of XPO1 with KPT-9058. KPT-9058 bound to XPO1 was extracted by streptavidin affinity from Jurkat wild-type or mutant cells. β-tubulin (loading control) and XPO1 were visualized by immunoblotting of the extract (top) and the total lysate (below) after 2h treatment with DMSO or 1 μM KPT-9058. **F.** Quantification of the XPO1 pull down with KPT-9058. The signal intensity (area under the curve) of the extracted XPO1 was quantified, divided by the signal intensity of β-tubulin and then compared to the wild-type. Bars represent means relative to the extraction from wild-type cells and error bars indicate standard deviation (N=2 repeated measurements of the same samples). The amount of XPO1 extracted from wild-type cells with DMSO is also shown for comparison. **G.** Quantification of cellular growth rates from parental wild-type or heterozygous and homozygous XPO1^C528S^ mutant cell lines. Cellular growth was analyzed over a period of 3-4 days and normalized to day 0. Data points represent means and error bars indicate the standard deviation (N=2, N=3 for Jurkat). Exponential growth curves were obtained by fitting with GraphPad Prism. **H.** Quantification of the cell diameter of wild-type and heterozygous or homozygous XPO1^C528S^ cell lines. Cell diameters were analyzed with a LUNA automated cell counter. Bars represent means and error bars indicate standard deviation (N=3).

In order to obtain cell lines carrying a cysteine to serine substitution at position 528 inside the XPO1 hydrophobic cargo-binding pocket, we applied CRISPR/Cas9 genome editing to alter the corresponding TGT DNA codon to TCA (Figure 1a). HL-60, Jurkat and K-562 leukemia cells were co-transfected with plasmids expressing Cas9-NLS and an *XPO1* targeting single guide RNA together with a 135 bases single-stranded oligodeoxynucleotide repair donor template containing the TGT to TCA mutation in addition to 3 silent mutations (Figure 1a). To enrich for cells that underwent homology directed repair with the 135 base oligo, transfected cells were treated with a relatively low dose of KPT-185 (Figure 1b) for 3 consecutive days. Only few cells survived (<10%), indicating a low efficiency of homology directed repair. The amount of surviving cells was highly dependent on transfection efficiency and cell-type, as more cells clearly survived in the easy to transfect K-562 and HL-60 cell lines (data not shown). Following transfection, single cells were distributed into 96-well plates to obtain single cell derived colonies. From these colonies genomic DNA was extracted and exon 15 of the *XPO1* gene was sequenced by Sanger sequencing. The majority of the clones integrated the desired missense mutation at only one of the *XPO1* alleles, while the other allele contained either the wild-type sequence, the silent mutations only, but not the desired missense mutation, or an insertion or deletion caused by non-homologous end-joining (NHEJ) (classified as hemizygous) (Figure 1c, Table 1). The remaining clones integrated the TGT to TCA mutation in both alleles. Two of the K-562 colonies only contained the wild-type sequence, suggesting that they tolerated the initial low dose selection with KPT-185. All sequences containing the desired missense TCA mutation also contained the three silent mutations, effectively ruling out spontaneous generation of resistance mutations during drug selective pressure.

**Table 1:** overview of the obtained mutant clones after CRISPR/Cas9 genome editing

XPO1			Cysteine_528_ substitution				Total Clones
Cell line	Leukemia	Substitution	Homozygous	Heterozygous	Hemizygous	Wild-type	
*HL-60*	**APML**	TGT_528_->TCA	21 (21.9%)	22 (22.9%)	53 (55.2%)	0 (0%)	**96**
*Jurkat*	**T-ALL**	TGT_528_->TCA	2 (12.5%)	10 (62.5%)	4 (25%)	0 (0%)	**16**
*K-562*	**CML**	TGT_528_->TCA	24 (33.3%)	11 (15.3%)	35 (48.6%)	2 (2.8%)	**72**

To confirm that these homozygous and heterozygous clones express mutant XPO1^C528S^ protein, wild-type XPO1 was extracted out of the cells with a biotinylated SINE compound, KPT-9058 (Figure 1d). KPT-9058 carries the typical acrylate-derived warhead required to bind cysteine_528_ of wild-type XPO1 [9] and it does not bind XPO1 protein containing the Cys528Ser mutation [12]. Wild-type, heterozygous and homozygous mutant cells were treated with KPT-9058 for 2 hours and KPT-9058 was then extracted from the cell lysate by streptavidin affinity purification. The co-precipitated XPO1 protein was detected by western blot analysis (Figure 1e). As expected, XPO1 protein was pulled down from wild-type cells, while it was impossible to extract XPO1 protein from the homozygous XPO1^C528S^ mutant cells. Quantification of the pulled down protein from heterozygous XPO1^C528S^ mutants showed that the amount of pulled down XPO1 protein was about half of that pulled down from wild-type cells (Figure 1f), confirming that half of the expressed XPO1 in the heterozygous mutants consists of wild-type protein while the other half consists of mutant XPO1^C528S^ protein.

The mutant cells appeared to grow slightly slower. To investigate this phenotype further, the cell proliferation of wild-type, heterozygous and homozygous mutants was analyzed over a period of 3-4 days (Figure 1g). A slightly slower growth rate in most of the mutant cells, and especially in the homozygous mutants, became apparent. More specifically, the growth rate was significantly lower in all Jurkat and HL-60 mutant cell lines when compared to the parental wild-type cell-line (p<0.0001). However, the growth rate of the heterozygous K-562 mutant cell line was not significantly different when compared to the parental wild-type (p=0.0968), but the homozygous K-562 mutant clearly did grow slower (p<0.0001). Finally, no significant differences in cell size between the hetero- and homozygous mutants and their respective parental wild-type cell line were observed (Figure 1h, p-values between 0.4 and 0.9).

### Heterozygous substitution of cysteine_528_ in XPO1 confers resistance to XPO1 inhibitors

**Figure 2:**
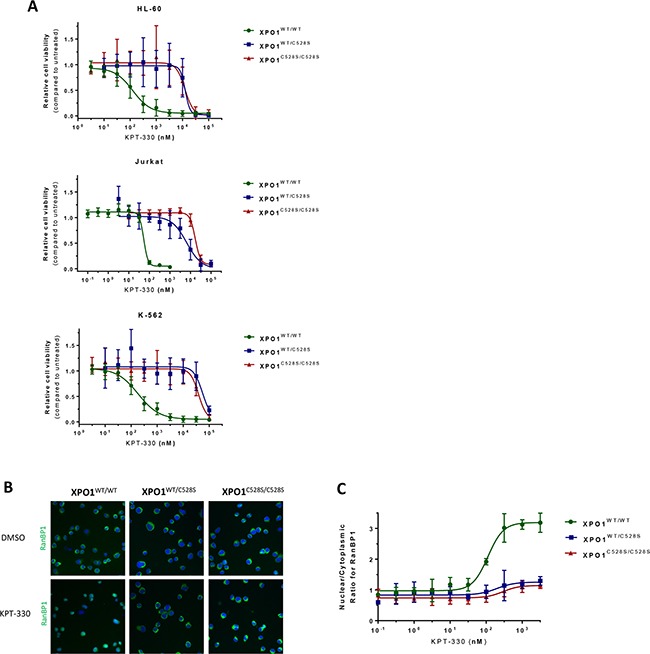
heterozygous substitution of cysteine_528_ in XPO1 by serine confers drug resistance **A.** Viability assays of the different cell lines treated with increasing concentrations of KPT-330 (selinexor) are plotted. Cell viability is expressed on the Y-axis as a fraction of the total response of untreated (DMSO) cells. The X-axis represents the drug concentration in nanomolar on a log_10_ scale. Points represent the average and error bars indicate standard deviation (N=3 duplicates). **B.** Visualization of the XPO1-mediated nuclear export of the cargo protein RanBP1. RanBP1 was visualized (green) with a high content imaging system by immunostaining of Jurkat cells treated for 3 hours with DMSO or increasing concentrations of KPT-330. Cell nuclei were counterstained with DAPI (blue). *Panel a* - visualization of the RanBP1 localization after treatment with DMSO. *Panel b* - visualization of the RanBP1 localization after treatment with 3 μM KPT-330. C. Quantification of the nuclear/cytoplasmic distribution of the XPO1 cargo protein RanBP1 as visualized by immunostaining. The Y-axis represents the ratio of the average nuclear signal divided by the average cytoplasmic signal on a cell per cell basis. The X-axis represents the drug concentration in nanomolar on a log10 scale. Points represent means and error bars indicate standard deviation (N=2).

To investigate whether resistance to selinexor (KPT-330, Figure 1b) is recessive or dominant for the cysteine_528_ mutation, hetero- and homozygous mutants, together with wild-type cells, were treated with selinexor. A remarkable reduction in cell viability of wild-type K-562, HL-60 and Jurkat cells was observed at nanomolar concentrations, while all the heterozygous and homozygous mutant cell lines were resistant to selinexor up to micromolar concentrations (Figure 2a; Table 2). Interestingly, there was no marked difference in resistance profile to selinexor between the heterozygous and homozygous mutants, demonstrating that the obtained genetic drug resistance to selinexor is dominant.

**Table 2:** overview of the EC_50_ values of selinexor obtained from the cell viability tests

Cell Line	EC_50_ (nM)		
	XPO1^WT/WT^	XPO1^WT/C528S^	XPO1^C528S/C528S^
*HL-60*	125.8 ± 22.5	12,683 ± 3,006	12,364 ±6,360
*Jurkat*	53.15 ± 3.6	6,490 ± 2,582	16,987 ± 1,052
*K-562*	183.7 ± 26.2	55,360 ± 13,836	38,575 ± 5,048

To confirm that a heterozygous Cys to Ser mutation in XPO1 is sufficient for selinexor resistance, we further examined the effect of selinexor on the XPO1-mediated nuclear export in heterozygous mutant cells. For this purpose, Jurkat cells were stained for endogenous RanBP1, which is a XPO1 cargo protein that is actively exported from the nucleus by XPO1 and localizes mainly to the cytoplasm in both wild-type and mutant cells (Figure 2b panel a). Upon treatment of wild-type cells with up to 3 μM selinexor for 3 hours, RanBP1 accumulated in the nucleus as a result from the inhibition of the XPO1-mediated nuclear export by the drug. In heterozygous mutant XPO1^C528S^ expressing cells, RanBP1 subcellular localization was unaffected upon selinexor treatment, demonstrating that a single heterozygous substitution of the XPO1 cysteine_528_ residue is sufficient for resistance against the SINE compounds (Figure 2b panel b). For each condition a minimal number of 200 cells were analyzed for the ratio of nuclear over cytoplasmic RanBP1 staining (Figure 2c). Remarkably, heterozygous mutants again showed a similar resistance profile as homozygous mutants. All in all, these findings further strengthen the conclusion that heterozygous mutation of the cysteine_528_ residue is sufficient for drug resistance.

## DISCUSSION

Exportin-1 (XPO1, CRM1) is a crucial protein involved in the nuclear export of many cellular proteins [13], including various tumor suppressive and growth regulatory proteins. Overexpression of XPO1 has been associated with various forms of cancer [2-6] and the inhibition of the XPO1-mediated nuclear export by small molecules has been shown to induce apoptosis and abolish cancer growth in *in vitro* as well as *in vivo* models of cancer [8, 9, 14]. The orally bio-available small-molecule XPO1 inhibitors called selective inhibitor of nuclear export (SINE) compounds, have proven anti-cancer activity in hematological and solid cancers, while sparing normal cells and showing limited off-target complications [7, 8]. Especially selinexor, the clinical candidate of this class of compounds, is showing promising results as single agent in patients with heavily pre-treated and relapsed hematological or solid cancer in multiple stage I/II/IIb clinical trials [15].

The drug has been shown to specifically and selectively interact with the cysteine_528_ residue located in the hydrophobic cargo-binding pocket of XPO1 [12]. Although this residue is conserved in higher eukaryotes, we generated multiple heterozygous and homozygous mutant leukemia cell lines containing a serine residue instead of the cysteine by using CRISPR/Cas9 knock-in genome editing. We demonstrated that the cys_528_ to ser substitution conferred cells with >100-fold resistance to selinexor. Using the biotinylated SINE compound KPT-9058 we showed that in heterozygous mutant cells, wild-type and mutant XPO1 are expressed in a 50/50 distribution. The XPO1^C528S^ protein expressed from the mutant allele in these heterozygous mutants is sufficient to elicit complete resistance to selinexor, demonstrating the dominant nature of this resistance mutation. Furthermore, this dominant resistance also demonstrates that 50% of the total XPO1 protein pool is sufficient for cellular homeostasis of the cell. However, the cysteine substitution appears to affect the cell proliferation slightly, which might be explained by an altered affinity of mutant XPO1^C528S^ to a subset of cargo-proteins, which in turn might alter the nuclear-cytoplasmic distribution of these XPO1 cargo proteins.

Little is known on possible resistance mechanisms to selinexor *in vivo*. Several studies focused on patient samples have suggested that recurrent mutation of residue E571 located in the NES binding groove of XPO1 might have prognostic or therapeutic value for different types of cancer [16-20]. However, this mutation does not alter selinexor sensitivity in primary mediastinal B-cell lymphoma [18]. Furthermore, a cryptic fusion of XPO1 with MLLT10 has been reported in T-ALL [21], but the effect of this translocation on selinexor activity remains to be investigated. Finally, the cysteine_528_ residue is highly conserved and mutation has not been observed in *in vivo* models. Additionally, selinexor is currently under investigation in many different clinical trials focused on its anticancer activity, but to our knowledge none of these trials have examined the appearance of cysteine_528_ mutations in the different treatment regimens. Nevertheless, here we have shown that heterozygous substitution of this single residue is sufficient to confer cells with resistance against selinexor. Interestingly, this mutation also resulted in decreased cellular growth rates, suggesting this mutation might reduce cancer cell fitness. However, further *in vivo* studies are needed to fully elucidate the possible effect of cysteine substitutions on cellular homeostasis, cancer cell fitness or tumor growth rate and selinexors anticancer activity.

To summarize, this study provides important predictive information of a possible resistance mechanism that could arise upon selinexor treatment. However, it remains to be seen whether a heterozygous or homozygous mutation of the XPO1 cysteine_528_ can arise in the clinic. Overall our results demonstrate that heterozygous genomic mutation of cysteine_528_ in XPO1 is sufficient for the development of a resistance mechanism to selinexor and further highlight the applicability of CRISPR/Cas9 genome editing for recessive and dominant drug-target interaction and validation studies.

## MATERIALS AND METHODS

### Cell lines

HL-60, Jurkat and K-562 cells were obtained from ATCC. All cells were cultured in 75cm^2^ flasks at 37°C and 4,5% CO_2_. Cells were grown in complete RPMI 1640 (Jurkat) or IMDM medium (HL-60, K-562) containing 2 mM L-glutamine and supplemented with 10% fetal bovine serum and 20 μg/mL gentamicin. Cells were kept between 3 to 15 x 10^5^ cells per mL.

### CRISPR/Cas9 knock-in

HL-60, Jurkat, and K-562 cells were transfected using electroporation with a Neon Transfection system (Invitrogen, Life Technologies). Briefly, cells were collected by centrifugation at 400*xg* and resuspended in Resuspension Buffer R (Invitrogen, Life Technologies) according to the instructions. Purified and highly concentrated DNA plasmids expressing Cas9-NLS (0.5 μg), single guide RNA targeting *XPO1* (0.5 μg) and the donor cys528ser oligonucleotide (1 μg) were added to the resuspended cells. Next the mixture, containing 200.000 cells, was electroporated with the following settings: 10 μL, 1350V, 10 ms and 3 pulses. Following electroporation, cells were immediately plated in 1 mL of antibiotic free RPMI 1640 or IMDM Medium (Gibco, Life Technologies) containing 10% fetal bovine serum in a 24-well plate and cultured at 37°C in a CO_2_ incubator. After two days the medium was refreshed and KPT-185 was added to a final concentration of 100 nM. Cells were then maintained and grown in the presence of the compound over a period of 1-3 weeks following standard cell culture guidelines. After this period, surviving cells were harvested and cultured further. When cultures were sufficiently grown, cells were plated at a density of 0.5 cells/well in 96-well plates in 20% FBS containing medium to obtain single cell derived colonies. Colonies were grown for 2-6 weeks and were regularly screened. Ultimately, colonies were harvested and the genomic DNA was extracted for Sanger sequencing.

### DNA constructs

The optimized sgRNA construct targeting *XPO1* near the cys528 coding region contains the following targeting sequence: 5′ GGATTATGTGAACAGAAAAG 3′-AGG (bold indicates the NGG protospacer adjacent motif). The oligonucleotide used for homologous recombination consisted of a 135 bases single stranded deoxynucleotide molecule containing two outer arms (50-80bp) homologous to the *XPO1* genomic region flanking the codon for cysteine528 of XPO1 and was synthesized by Integrated DNA Technologies. The oligonucleotide contained three silent mutations near the PAM site and two point mutations at the cys528 coding triplet to provide the template for the Cys528Ser mutation and consisted of the following sequence: 5′-GCTAAATAAGTATTATGTTGTTACAATAAATAATACAAATTTGTCTTATTTACAGGATCTATTAGGATTA**TC**AGAACAGAAgcGcGGCAAAGATAATAAAGCTATTATTGCATCAAATATCATGTACATAGTAGG-‘3 (bold indicates the cys528ser missense mutation, lowercase and underlined indicates additional silent).

### DNA extraction and sequencing

When single cell derived colonies in 96-well plates were sufficiently grown, cells were washed and then lysed in Bradley lysis buffer at 56°C (10 mM Tris-HCl (pH 7.5), 10 mM EDTA, 0.5% SDS, 10 mM NaCl and 1 μg/mL proteinase K). The genomic DNA was extracted from the lysate using ethanol-salt precipitation coupled to centrifugation. The target site, the DNA sequence around *XPO1*, was amplified by PCR with the following primers: fwd: 5′-TCTGCCTCTCCGTTGCTTTC, rv: 5′-CCAATCATGTACCCCACAGCT. After PCR, the products were sequenced by Sanger Sequencing (Macrogen) using a forward and reverse primer (5′-TGTGTTGGGCAATAGGCTCC, 5′-GGCATTTTTG GGCTATTTTAATGAAA respectively).

### XPO1 pull down with KPT-9058

For pull down of XPO1 out of cells using KPT-9058, 5x10^6^ Jurkat cells were plated in 1.5 mL of RPMI supplementend with 2 mM L-glutamine, 10% FBS and 20 μg/mL gentamicin inside 6-well plates. After a few hours inside the incubator, 1.5 mL of RPMI containing 2 μM of KPT-9058 was added to the cells (final concentration 1 μM). Cells were incubated for 2 hours and then collected by centrifugation (400x*g*) and washed in ice-cold PBS. Cell pellets obtained after centrifugation (400x*g*) were lysed on ice in RIPA buffer supplemented with 1x HALT protease inhibitors (Thermo Scientific) and then cleared from debris by centrifugation at 20.000x*g* for 10 min at 4°C. Protein concentrations were measured using a colorimetric BSA protein assay (Pierce). A fraction of the cell lysis mixture was taken for quantification of β-tubulin and XPO1 total protein. The rest of the extracts were allowed to bind to Dynabeads MyOne Streptavidin T1 (Life Technologies) by rotating overnight at 4°C in order to capture KPT-9058. Following overnight incubation, the beads were washed 5 times in modified RIPA buffer (50 mM Tris-HCl pH 7.8, 150 mM NaCl, 1% NP-40 (IGEPAL CA-630), 0.1% sodium deoxycholate, 1 mM EDTA) and subsequently boiled for 10 minutes in 0.5% SDS containing 1x sample buffer (Protein Simple) to elute captured proteins. Proteins were finally separated by size (12-230 kDa) and visualized on a Wes system (Protein Simple) with an anti-rabbit HRP conjugated antibody detecting the primary XPO1 (1/12500, NB100-79802) and β-Tubulin (1/3000, NB600-936) rabbit antibodies. Protein signals were visualized and quantified with the Compass software, v2.7.1 (Protein Simple).

### Cell growth and size assays

Growth rates of mutant and wild-type cell lines were determined with the CyQuant Direct Cell Proliferation kit (ThermoFisher Scientific). Initially a standard curve was obtained to determine the relation of the fluorescent signal to the amount of cells present in the sample. For the experiment, 20.000 Jurkat cells or 10.000 K-562 and HL-60 cells were seeded in different 96-well plates on day 0 in complete RPMI or IMDM medium respectively. The cells were allowed to grow over a period of 3-4 days and the amount of cells was determined every 24 hours by measuring fluorescence of the CyQuant Direct Cell Proliferation reagent with a SaFire II microplate reader according to the manufacturer's instructions. The obtained data points were compared to the previously obtained standard curves and cell numbers were normalized to day 0. Growth curves were then obtained by fitting the data with an exponential growth curve in GraphPad Prism and statistical significance was determined. The experiment was performed in triplicate and repeated at least once. For the determination of average cell sizes, all cells were analyzed during 3 different passages with the LUNA automated cell counter (Logos Biosystems). Statistical significance between cell sizes was determined with ANOVA.

### Cell viability assays

Cell viability assays were performed by plating 10.000-20.000 cells in 96-well plates containing DMSO or a dilution of KPT-330. Cells were incubated for 72 hours at 37°C and 4.5% CO_2_. Cell viability was assessed with the CellTiter 96® AQueous Non-Radioactive Cell Proliferation Assay reagent (Promega) according to the manufacturer's instructions. Absorbance of the samples was measured at 490 nm using a SaFire II microplate reader (Tecan). All assays were performed in duplicate and each experiment was repeated at least three times. The data was analyzed using a log-based 4 parameter model in GraphPad Prism and the bottom value for the EC_50_ calculation was set by hand for the mutant K-562 cells.

### High content imaging of RanBP1

Jurkat cells were plated in a 96-well imaging plate (Falcon) and treated with a dilution series of selinexor or carrier (DMSO) for 3 hours. After treatment, cells were washed with PBS and fixated for 10 minutes in 4% PFA. The cells were then washed and remaining PFA was inactivated with PBS with 0.1M glycine. Cells were permeabilized with PBS containing 0.1% Triton X-100 for 10 minutes. Subsequently, the cells were washed 3 times with PBS and then blocked at 37°C for 2 hours in 10% Normal Goat Serum. The primary anti-human RanBP1 (Ab97659, Abcam) antibody was then added and incubated for 1 hour. After primary staining, the cells were extensively washed and the secondary antibody, Alexa Fluor 488 (A11008, Invitrogen), was incubated for 1 hour in the dark. Finally, cells were washed and the nuclei were counterstained with DAPI. The plate was imaged using the green and blue channels of an ArrayScan XTI High Content Reader (ThermoFisher Scientific). Nuclear and cytoplasmic compartments were segmented and their average pixel intensities were quantitated with the HCS Studio software. The obtained nuclear/cytoplasmic ratios were analyzed with GraphPad Prism. The experiment was repeated two times.
